# As pesquisas constitucionais no entreguerras, 1922

**DOI:** 10.1590/S0104-59702024000100041

**Published:** 2024-10-11

**Authors:** Renilson Beraldo

**Affiliations:** i Doutor, História das Ciências e da Saúde/Casa de Oswaldo Cruz/Fiocruz. Rio de Janeiro – RJ – Brasil beraldo_rfs@hotmail.com

**Keywords:** Constituições, Fonte primária, Pediatria austríaca, Meinhard Pfaundler (1872-1947), História da Medicina, Constitution, Primary source, Austrian pediatrics, Meinhard Pfaundler (1872-1947), History of medicine

## Abstract

O documento aqui apresentado é uma tradução comentada do artigo “Was nennen wir Konstitution, Konstitutionanomalie und Konstitutionskrankheit?” [O que denominamos constituição, anomalia constitucional e doença constitucional?], publicado pelo pediatra germânico Meinhard Pfaundler em 1922. Encomendado pelo Klinische Wochenschrift [Semanário Clínico], o artigo tinha por objetivo apresentar e aclarar as mais recentes discussões e terminologias a respeito da “doutrina constitucional” em medicina. O artigo “Was nennen wir Konstitution...”, portanto, fornece dados teórico-conceituais a respeito do debate constitucional no circuito médico europeu no período entreguerras.

## Abstract (en)

The article “Was nennen wir Konstitution, Konstitutionanomalie und Konstitutionskrankheit?” [What do we call constitution, constitutional anomaly, and constitutional disease?], published in 1922 by the German pediatrician Meinhard Pfaundler, is presented in an annotated translation into Portuguese. Commissioned by Klinische Wochenschrift [Clinical Weekly], the article presents and discusses what were at the time the most recent debates and terminology concerning the “constitutional doctrine” in medicine. “Was nennen wir Konstitution...” therefore sheds theoretical and conceptual light on debates regarding the constitution in European medical circles in the interwar period.

Na virada do século XIX para o XX, as transformações na noção de doença a partir de sua especificidade (etiologia monocausal externa ao indivíduo) foram resultado de negociações institucionais, burocráticas, intelectuais e técnicas que podem ser comparadas a uma revolução cultural. Conforme Rosenberg (2007, p.17), as doenças como categorias são fundamentais para a interlocução entre conhecimento e prática, bem como para compreender a extensão dos territórios idiossincráticos (predisposições individuais) em relação às intervenções práticas generalizantes (intervenção vacinal, por exemplo). Trata-se, nesse sentido, de dar atenção à historicidade de noções como saúde, doença, diagnóstico, prognóstico, terapêutica, organismo etc.

O exame histórico do conceito médico de “constituição” permite-nos interrogar a quase totalidade dessas noções. Expressão dos territórios idiossincráticos, o estudo das constituições foi um dos objetos prestigiados pela medicina clínica no início do século XX. Tal prestígio teria sido abafado por um longo período após a generalização de métodos de localização dos germes com a bacteriologia, a microbiologia e a experimentação laboratorial (Vácha, 1985). É verdade que o fenômeno da constituição e todo léxico a ele correspondente não podem ser vistos como algo estanque que teria sido “despertado” apenas no início do século XX, conforme demonstra Beraldo (2021). Na realidade, a lacuna aberta pela incompletude das explicações etiológicas analíticas e reducionistas, focalizando a busca pelos agentes causadores de doenças e infeções, abriu espaço para que os elementos integrantes da vida (saudáveis ou patológicos) fossem explicados pelo fator constituição: “A revolução bacteriológica na medicina foi igualmente a sua revolução constitucional” (Mendelsohn, 2001, p.26-35).^1^

O interesse da medicina moderna nas constituições foi atualizado entre o final do século XIX e o início do XX. Ou seja, determinados aspectos dessa doutrina fazem parte do pensamento médico há muito tempo; essa longa duração foi acionada frequentemente por médicos de diversos locais para legitimar tal doutrina. Foi a partir de permanências do léxico constitucional na prática médica que tal pensamento pôde ser atualizado no início do século XX, no interior de um materialismo mecanicista, no qual a teoria médico-científica, pautada na *Entwicklungsmechanik* [mecânica do desenvolvimento] – de Wilhelm Roux, 1850-1924) – reportava-se aspectos físico-químicos para explicar as causas dos fenômenos biológicos (Allen, 2017, p.100-106; Canguilhem, 2012, p.89-90; Nicholson, 2010, p.46-51).

Com base na referida longa duração, alguns aspectos do vocabulário e dos conceitos constitucionalistas podem ser encontrados no *Corpus Hippocraticum* e na patologia humoral galênica (Ackerknecht, 1982, p.319-320). A obra hipocrática é uma variada reunião de textos diferenciados, e considera-se que nem todos sejam de autoria de Hipócrates.^2^ Sob o ponto de vista hipocrático (“escola de Cós”), o material clínico visado pelo pensamento médico pautava-se na tipificação dos doentes, e não das doenças, enquanto, para a “escola de Cnido”, as nosologias seriam o ponto de partida da prática médica (Pinillos, Piñero, Ballester, 1966, p.18-19). De certa maneira, a exigência hipocrática de que o olhar estivesse voltado para o doente, e não para as doenças, permeou o constitucionalismo do início do século XX. Essa passagem da doença para o doente, ou seja, a prática médica orientada constitucionalmente, possibilitará um recuo no tempo para pensar o vivente e sua história por trás da doença.

A medicina hipocrático-galênica estava organizada em torno de quatro humores: sangue (quente), fleuma (frio), bílis amarela (seca), bílis negra (úmida). A prevalência, o excesso ou o exagero de um ou outro desses humores poderia definir os temperamentos, também em número de quatro: melancólico, colérico, sanguíneo e fleumático. Esses seriam os tipos constitucionais conforme o pensamento clássico (Timmermann, 1996, p.17-18). O conceito de constituição a partir da escola coica serviu como um instrumento auxiliar da prática médica, fundamentalmente centrado no prognóstico, já que um tipo constitucional específico poderia “predispor” a um determinado abatimento (Pinillos, Piñero, Ballester, 1966, p.32). Somente um “equilíbrio” das propriedades, descritas acima, poderia proporcionar a saúde.

Ao longo do tempo, o pensamento médico acionou um conjunto terminológico variado para falar da constituição. Distintos termos aparecem nos tratados atribuídos aos coicos, como no livro *Epidemias*, nos *Aforismos*, nos *Prognósticos*, entre outros, que acabaram conformando o *Corpus hippocraticum*. O ponto de partida da escola coica era considerar o indivíduo em relação com o meio ambiente (natureza e costumes/normas). As distintas modalidades dessa relação conformariam tipos que, por sua vez, poderiam predispor a enfermidades peculiares. Ainda não estamos no espectro amplo da teoria humoral (os quatro humores), apenas no tratado tardio *De natura hominis* tal sistematização aparecerá, sobretudo, a partir de Galeno (Pinillos, Piñero, Ballester, 1966, p.34).

Olhar para o conjunto terminológico é importante, tendo em vista os fluxos e influxos desse problema na história da medicina. Em tais tratados, o termo *katástasis* referia-se à constituição, mas essa referência significava os “tipos climatológicos”, ou seja, uma dada “figura corporal” resultante da relação do indivíduo com a natureza (*physis*). A “figura corporal”, por sua vez, era resumida no grego pelo termo *eidea/eidos* (noção de imagem). Em substituição a *eidea/eidos,* Galeno insere o termo *exis* para “figura corporal” (traduzido para o latim como *habitus*) (Pinillos, Piñero, Ballester, 1966, p.35-36). Por fim, o termo grego *krasis*, utilizado com mais frequência por Galeno para referir-se à perfeita adequação dos quatro humores, tornar-se-á *temperamentum* (Ackerknecht, 1982, p.319).

Em meados do século XIX, ocorrera a ênfase mais ou menos pronunciada sobre um ou outro conceito do conjunto lexical aqui descrito. Naquele contexto, as variações de estrutura interna dos órgãos (vísceras, sistemas, aparelhos), sua relação com anomalias e potenciais enfermidades foram estudadas por Claude Sigaud (1862-1921), Achille De Giovanni (1838-1916) e Friedrich Wilhelm Benecke (1824-1882), detendo-se apenas nos aspectos físicos e na estrutura anatômica dos indivíduos (visada morfológica, ênfase no *habitus*). Eram anatomistas lidando com mensurações de cadáveres, em cujas abordagens o aspecto funcional e fisiológico, por exemplo, estava ausente. Por outro lado, o programa de pesquisa dos constitucionalistas das primeiras décadas do século XX diferenciava-se do de seus antecessores por inserir o fator “paciente vivo” em contraste aos “corpos mortos” (Timmermann, 1996, p.30-31). Com o paciente vivo, o aspecto psicossomático pôde ser inserido. Nessa seara, as disputas, muitas vezes, estarão circunscritas na diferenciação do papel maior ou menor do genótipo ou do fenótipo como condição ou expressão da constituição.

Ainda um adendo à discussão terminológica: o termo *katastasis*, traduzido para o latim como *constitutio*, denotou, até o final do século XIX, dois aspectos: “constituição individual” e “tipo meteorológico”, sinalizando, assim, para a prolongada e tortuosa relação histórica entre o indivíduo e o meio externo. Essa relação está muito bem expressada nas edições do dicionário de Émile Littré (1801-1888)^3^ e Charles Robin (1821-1885), cânone da medicina clínica europeia do século XIX. Da décima edição (1855), passando pela décima segunda (1865), até a décima terceira (1873), percebe-se a permanência não apenas do termo *katastasis* (κατάστασις, no grego antigo), mas da definição de “constituição” como “montagem de várias partes que formam um todo”^4^ e “estado geral da organização particular de cada indivíduo”^5^ (Littré, Robin, 1855, p.319-320; 1865, p.344-345; 1873, p.346-347).

Apesar de manter em suas definições a ideia de “estado geral de organização particular de cada indivíduo” (formado pelas vísceras, sistemas e aparelhos) e sua relação com influências atmosféricas (“constituição atmosférica”), enfermidades reinantes em um determinado local (“constituição médica”), epidemias (“constituição epidêmica”) e estações do ano (“constituição sazonal”), Littré inserirá uma mudança significativa a partir da 16ª (1886) e 21ª (1905) edições. Nelas, afirmará que as distintas constituições (resultantes da referida falta de equilíbrio correspondente aos sistemas) poderiam ter “*une grande influence sur le développement la nature et la marche des maladies, sur le pronostic que celles-ci comportent, sur le traitement qu’elles exigent*” [uma grande influência no desenvolvimento, na natureza e no curso das doenças, no prognóstico que elas acarretam, no tratamento que requerem] (Littré, 1886, p.360; 1905, p.378). Ou seja, a ênfase, nessa definição do final do século XIX, é na caracterização da constituição como elemento importante para o prognóstico e seu tratamento.

Resumidamente, com poucas diferenças, Littré parece estabelecer, em suas definições, em primeiro lugar, uma concepção unitária e totalizante da constituição (montagem de várias partes que formam um todo); em segundo, o seu estado de “forte-fraca” a depender da “vitalidade” e do “equilíbrio” dos sistemas e das funções; em terceiro, uma definição ambiental (atmosférica, médica, epidêmica, sazonal). Décadas depois dessas definições, como veremos, Pfaundler encontrará o programa da medicina constitucional inserido (ainda) nessa recorrência relacional entre fatores individuais (fatores genotípicos) e ambientais (fatores paratípicos), embora em chave médico-teórica bastante distinta.

No contexto do renascimento do neo-hipocratismo e neovitalismo na França e na Alemanha do início do século XX (Waisse, Amaral, Alfonso-Goldfarb, 2011), o estudo das constituições trazia a marca significativa da orientação de uma medicina voltada para o organismo e suas condições pessoais. Conforme Timmermann (1996, p.45-46) e Lawrence e Weisz (1998, p.6), no contexto germânico, o filósofo Hans A.E. Driesch (1867-1941) foi um dos principais articuladores do neovitalismo para as ciências da vida, tendo ainda discutido questões relacionadas à noção de pessoa como unidade psicofísica. Essa questão foi suscitada num contexto de teorias parasitárias, quando os médicos questionavam a “receptividade” do paciente para adquirir uma doença, fosse específica ou não (Contrepois, 2002, p.200). Para o estabelecimento de uma patologia, afirmava-se a necessidade não apenas de um agente infeccioso, mas também da variabilidade particular de reação do paciente para estar ou não doente, ou melhor, do “grau de suscetibilidade dos indivíduos à doença” (Vácha, 1985, p.340). Tais particularidades, suscetibilidades e predisposições foram traduzidas a partir de alguns conceitos, a exemplo do conceito de “constituição”.

## O conceito de constituição no texto de Pfaundler, de 1922

A definição do conceito de constituição esteve envolvida em concordâncias e controvérsias, tanto em textos de médicos brasileiros como de outros países (Beraldo, 2021). No contexto linguístico germânico das primeiras décadas do século XX, o debate sobre o conceito de constituição foi uma pauta de interesse comum entre os médicos, embora sem a devida concordância a respeito de seu conteúdo. O número de artigos com a entrada *Konstitution* e termos correlatos, como *Konstitutionslehre* [doutrina constitucional], *Konstitutionsanomalie* [anomalia constitucional] e *Konstitutionskrankheit* [doença constitucional], publicados nos principais jornais médicos alemães entre 1900 e 1938, teve um incremento sobretudo entre 1921 e 1930 (Timmermann, 2001, p.722-723). Foi precisamente no período entreguerras que Meinhard von Pfaundler (1872-1947) publicou o artigo “O que denominamos constituição, anomalia constitucional e doença constitucional?” (Pfaundler, 22 abr. 1922).

Meinhard Pfaundler von Hadermur foi um pediatra, nascido em Innsbruck (Áustria), local onde iniciou sua formação em medicina. Na Universidade de Graz, estava entre seus professores o internista Friedrich Kraus (1858-1936), importante teórico dos estudos constitucionais, conforme veremos adiante. Na referida universidade, Pfaundler começou a atuar como médico assistente de clínica infantil (1896). Nesse período, publicou trabalhos sobre sorodiagnóstico, fisiologia e metabolismo de doenças em crianças. Posteriormente, em 1902, tornou-se professor e diretor do Hospital Infantil da Universidade de Viena. Em 1906, transladando-se para a Alemanha, passou a dirigir a Clínica Infantil da Universidade de Munique, obtendo também uma cátedra para ensino e formação a partir de 1912 (Timmermann, 1996, p.73-74). Quando estava em Munique, Pfaundler publicou o *Manual pediátrico* (quatro volumes) focalizando a semiologia de doenças e em coautoria com Arthur Schlossmann (1867-1932), referência em doenças infantis no período. Portanto, no que se refere ao contexto institucional de produção do texto aqui apresentado, Pfaundler estava na supracitada clínica em Munique, já havia publicado inúmeros trabalhos, sobretudo a respeito de nutrição infantil e aspectos sociais do exercício da pediatria (educação, higiene, cuidados) (Wormer, 2001, p.303-304).^6^

O artigo de Pfaundler é relevante por dois motivos: trata-se de uma síntese das discussões e concepções a respeito das constituições até aquele momento, realizada a convite do semanário médico *Klinische Wochenschrift*; o autor também buscou localizar as mudanças na *Konstitutionsforschung* [pesquisa constitucional] desde o início do século XX. Para tal, relacionou interpretações empregadas pela escola de Berlim-Rostock por Friedrich Martius (1850-1923), Friedrich Kraus (1858-1936) e Theodor Brugsch (1878-1963) e pela escola de Viena por Julius Tandler (1869-1936) e Julius Bauer (1887-1979), entre outras.

Com intenção de pautar o debate e não de resolver o problema, Pfaundler abre o artigo enfatizando que em nenhuma outra área da patologia clínica reinava confusão tão irremediável como na área da pesquisa constitucional. A seguir, traça um panorama do que ele visualizava nas abordagens médicas sobre o tema naquele contexto de início da década de 1920. De acordo com Pfaundler, a primeira, e uma das concepções mais antigas, era a de que a noção de constituição seria tudo o que se disseminava no corpo e não possuía localização definida. Essa concepção foi chamada por ele de “localística”. Em uma segunda concepção, o que demarcaria um caráter constitucional seria a “duração de um estado” corporal; por exemplo, um indivíduo que fosse, durante muito tempo, portador de sífilis e cujos processos vitais sofressem alterações substantivas, locais e gerais (constituição sifilítica). Uma terceira fazia menção à constituição como sendo “fraca” ou “forte”, isto é, quando as características corporais estivessem respectivamente sub- ou sobrevalorizadas em relação à média (Pfaundler, 22 abr. 1922, p.817).

Ainda em conformidade com Pfaundler, para alguns clínicos a constituição teria um aspecto dinâmico: constituição como fluxo [*Ablauf*], ou seja, uma “forma de reação”*.* Para outros, a constituição seria mais como uma estrutura [*Anlage*] e potência: uma “capacidade de reação”. Nesta última interpretação (*Anlage*), a constituição também era descrita como uma “soma de características” ou “complexo de sintomas”. Pfaundler buscou na etimologia da palavra constituição a seguinte caracterização terminológica: compilação [*Zusammenstellung*], composição [*Zusammensetzung*], constituição [*Verfassung*], condição [*Beschaffenheit*], ordenamento [*Anordnung*] ou organização [*Einrichtung*]. É interessante que Littré também utilize *Beschaffenheit* para referir-se à constituição no idioma germânico.

Conforme observamos no texto de Pfaundler, a definição do conceito de constituição entrava em embate com a noção de anomalias constitucionais, as quais, por sua vez, eram empregadas no contexto germânico por diferentes médicos, embora existissem dúvidas a respeito de sua operacionalidade. As indefinições das anomalias constitucionais pertencem à mesma trama conceitual das diáteses, assim como das disposições na história do pensamento médico. Ackerknecht (1982) indica que, em 1911, no Congresso Alemão de Medicina Internacional, três autores apresentaram relatórios sobre o tema das diáteses: W. His, Bruno Bloch e Pfaundler. Os três teriam respaldado a noção de diátese, debatendo, entre outras, a diátese exsudativa, eosinofílica e astênica (Ackerknecht, 1982, p.324). Para o referido autor, os estudos sobre alergia, endocrinologia e bacteriologia foram preparando o caminho para uma mudança de sentido das diáteses. Tal mudança possibilitou a permanência, nos anos seguintes, de questões presentes no âmbito das diáteses em seu sentido mais antigo como “arranjo”, “disposição” ou “condição”. Assim, na década posterior, “os adeptos das disposições marcharam sob a bandeira da constituição” (p.324).

Ao longo da década de 1920, portanto, a ciência da constituição seguiu em “estado florescente”, conforme asseverou o médico internista vienense Julius Bauer (1928, p.127). Além de Bauer, também F. Kraus e F. Martius assentiam que, para além do diagnóstico da doença, a prática médica rotineira deveria pressupor a “individualização” na análise diagnóstica. Tal individualização, ou melhor, a passagem do diagnóstico da doença para o diagnóstico do doente, seria alcançada pela aferição das características constitucionais do indivíduo. Martius, por exemplo, conhecido no período entreguerras como um dos pioneiros da medicina constitucional e responsável por sua popularização, buscou compreender o porquê da diferença expressa no curso de uma mesma doença em diferentes indivíduos (Timmermann, 2001, p.721-722). Tal diferença implicava, para aqueles clínicos, apreender e suspeitar da própria relação médico/paciente e do paciente com a sua enfermidade.

A concepção constitucional mais adequada a Pfaundler (embora admita ser incerta) seria resultado da determinação oscilante de fatores endógenos (fatores genotípicos) e ambientais (fatores paratípicos). Aqui, o nome do anatomopatologista Carl Hart (1876-1922) é central para a discussão sobre até que ponto a constituição individual seria manifestação do genótipo ou do fenótipo. Hart e Bauer, citados no artigo de Pfaundler, estão envolvidos criticamente com a definição de constituição como “destino somático do indivíduo”. Tal definição remonta ao anatomista e ginecologista Paul Mathes (1871-1923) e Julius Tandler, que, via Áustria, desde a segunda década do século XX, articularam essa forma de entender a constituição individual.

Nesse sentido, para alguns clínicos, como Bauer e Tandler, a constituição seria uma realização, um resultado da potência de “fatores endógenos” – expressão ainda um tanto incompreendida e polissêmica no período. Para outros, como Martius, Kraus e Brugsch, o conceito de constituição não era baseado apenas no genótipo. Uma constituição poderia ser adquirida, assim como a imunidade a certas doenças. Theodor Brugsch, outro aluno de Kraus, propôs uma equação para constituição segundo a qual o equilíbrio entre fatores internos (genotípicos) e externos (ambientais) seria o objetivo final do organismo para se manter vivo (Timmermann, 1996, p.27), sua capacidade de reação. Tal definição de constituição era suficientemente aberta para permitir a inserção de observações voltadas para o social e sugerir constituições criminais, tuberculosas, psiquiátricas etc.

Brugsch, Martius, além de Gustav von Bergmann (1878-1955), conformaram a escola de R. Kraus de pesquisa constitucional na Charité de Berlim. Em suas pesquisas, referiam-se à ideia de totalidade da pessoa (*Personalismus*), bem como à “patologia funcional” (Beraldo, 2021; Timmermann, 1996). Pfaundler corrobora a posição desses autores. Assim, aproximando-se da escola de Berlim (lembrando que ele havia sido aluno de Kraus), deixa evidente sua crítica à falta de utilidade prática (na clínica) do conceito de constituição baseado em fatores endógenos imensuráveis.

As dificuldades em apreender o conceito de constituição por meio da interpretação genotípica e hereditária fizeram com que Pfaundler, tendendo a uma definição mais fenotípica da constituição, afirmasse o seguinte: um conceito médico como o de “constituição” só teria valor se pudesse ser efetivamente observado na prática clínica. Nesse sentido, as noções contrastantes de constituição, apontadas por Pfaundler, demonstram como os conceitos médicos são objetos negociados nos espaços tanto da prática clínica quanto no meio médico associativo. Um exemplo disso é que o referido autor advogava para a Sociedade Alemã de Medicina Interna [Gesselschaft für innere Medizin] a iniciativa de determinar as definições e nomenclaturas mais relevantes a respeito daquelas noções.

O artigo de Pfaundler evidencia ainda que os adeptos do conceito de constituição não abandonaram inteiramente o conceito de disposição. Nessa leitura, o fator constitucional como meio de compreensão do *Orbis morborum* [mundo das doenças], na acepção de Pfaundler, permeou distintos campos disciplinares, como a medicina, a embriologia, a fisiologia, entre outros. O esforço de Pfaundler explicita apenas um dos capítulos de uma história mais ampla de tentativas, por parte da medicina, de definir os esquemas causais das doenças. Em tal capítulo, as discussões mobilizaram categorias explicativas do ser humano em seu passado, presente e futuro patológico.

Por fim, vale ressaltar que, ao mirar o conceito de constituição, veremos seu emprego articulado a discussões mais gerais, como a rejeição da bacteriologia como “bala mágica”; a defesa da totalidade do organismo como contraponto à sua fragmentação físico-química pelo mecanicismo; sua utilização em elaborações eugênicas, bem como a criação de tipologias para remeter a aspectos físicos e psicológicos no contexto da biotipologia da década de 1930 (Vimieiro-Gomes, 2016; Beraldo, 2016). Vale ressaltar, no entanto, que esse programa de pesquisa, atribuído, sobretudo, ao nome de Nicola Pende (1880-1970), aparecerá em outro contexto linguístico, político e institucional, apesar de ter se nutrido das discussões ocorridas no idioma germânico nos anos anteriores.^7^ Por isso a ênfase em trazer, neste texto, um debate distinto do recorrente na historiografia correlata, que tende a subsumir a análise histórica do léxico constitucional à biotipologia (resenhada como tipologia constitucional) em um contexto totalitário. O artigo de Pfaundler, publicado no começo da década de 1920, localiza-se, portanto, em uma encruzilhada entre as concepções de constituição elaboradas por clínicos da década anterior e o que viria a ser a galvanização do conceito sob as orientações fascistas das décadas seguintes.

A seguir, o leitor encontrará o documento-artigo “Was nennen wir Konstitution...”, em sua versão traduzida e inédita; até o momento, não existia registro de outra versão além da publicada no idioma germânico, em 1922.

## NOTAS

1 A historiografia relacionada utiliza a noção “constitucionalismo” intercambiando-a com outras, como: medicina constitucional, teoria constitucional, pesquisa constitucional e doutrina constitucional. No artigo de Pfaundler, encontram-se os termos *Konstitutionsforschung* [pesquisa constitucional] e *Konstitutionslehre* [doutrina constitucional]. Tais noções, portanto, são constructos de bibliografias secundária e primária. Ver, entre outros: Vácha (1985); Timmermann (1996, 2001); Lawrence e Weisz (1998).

2 Hipócrates foi um médico grego que viveu por volta do século V a.C. O nome do seu local de nascimento, a ilha de Cós, serviu, historicamente, para identificar a “escola de Cós” (ou escola coica) em contraposição à “escola de Cnidio” (Pinillos, Piñero, Ballester, 1966, p.18-19).

3 Filósofo e lexicógrafo francês, responsável pela edição de dicionários de terminologia médica e ciências afins, bem como pela tradução das obras completas de Hipócrates.

4 No original: “*Assemblage de plusieurs parties qui forment un tout*”.

5 No original: “*État général de l’organisation particulière de chaque individu*”.

6 Conforme Wormer (2001), no contexto do nacional-socialismo, Pfaundler demonstrou abertamente sua rejeição ao regime nazista. Por isso seu nome não fora incluído do panteão da Academia de Ciências da Bayer em 1941. Os dados dos demais autores citados constam nas notas anexas ao documento comentado e apresentado ao final do presente texto.

7 Nicola Pende fez seis meses de especialização em 1914 na Charité de Berlim. Esse circuito necessita ser investigado. Para as apropriações das escolas alemã e italiana na medicina brasileira, ver, respectivamente: Beraldo (2021); Vimieiro-Gomes (2016).
