# Supplementary material for: Research into the constitution in the interwar period, 1922
Source: Hist Cienc Saude Manguinhos. 2024 Oct 11;31:e2024041. [Article in Portuguese] doi: 10.1590/S0104-59702024000100041 (PMC11473110; doi:10.1590/S0104-59702024000100041)
Supplement: Related texts

# Reprint: Klinische Wochenschrift [Semanário Clínico]

**Authors:** M. Pfaundler

**Affiliations:** Diretor da Clínica Pediátrica Universitária em Munique

Ano 1 – n.17 – 22 de abril de 1922

## SÍNTESE

O que denominamos constituição, anomalia constitucional e doença constitucional?^1^

É provável que em nenhuma outra área da patologia clínica reine confusão tão irremediável como na área mencionada no título. Inúmeras contribuições valiosas sobre o assunto, em tempos recentes, não modificaram essa situação. E cada vez fica mais claro que, por falta de uniformidade sobre a definição dos conceitos, as discussões são infrutíferas. Novas ideias importantes sobre a teoria das constituições fisiológicas ou patológicas não são levadas em conta ou não são compreendidas, porque o autor fala uma língua completamente diferente da de seus leitores ou ouvintes. Essa queixa, feita também por muitos outros,^2^ de *impedimento do progresso, faz com que seja premente e desejável que surja finalmente um acordo – o mais amplo possível – sobre os conceitos fundamentais*.^3^ Só então poderão todos ver com clareza se e por qual motivo as teorias dos diversos pesquisadores não coincidem, e somente assim se chegará a poder colocar questões precisas. *Minha intenção é oferecer um incentivo para que a Sociedade Alemã de Medicina Interna, como órgão autorizado, inicie a determinação das definições e nomenclaturas mais relevantes de forma adequada*. Entre os sócios dessa instituição se encontram os mais renomados conselheiros, que certamente estariam prontos a defender uma boa causa e que, sem sombra de dúvida, poderiam chegar a um consenso entre si, sem por isso ter que abandonar seus pontos de vista subjetivos.

Talvez seja possível, na continuidade, apontar alguns dos aspectos mais atuais dessa discussão cada vez mais acirrada; não se pretende esmiuçar todo o problema, o que, além do mais, já foi feito de forma excelente por Martius,^4^ em especial da perspectiva histórica e genética.

Inicialmente consideremos três concepções mais antigas sobre o caráter das constituições e suas anomalias. Uma delas é a concepção de que constituição é tudo o que se dissemina no corpo de maneira geral e que não apresenta alguma localização definida. O quão pouco se poderia fazer com essa *definição localística* em termos de uso da linguagem e das acepções correntes pode ser percebido se pensarmos em envenenamentos ou infecções agudas e amplamente disseminadas, que jamais alguém considerou “constitucionais”. A teoria triunfante das constituições parciais (Martius) também invalida completamente esse entendimento.

A segunda é a concepção de que *a duração de um estado* seja determinante para marcar o caráter constitucional. Sobre isso (e também sobre o princípio da localização), diz, por exemplo, Virchow:^5^ “Uma doença se torna constitucional quando *durante um longo período de tempo* a direção dos processos vitais se desvia do normal, do habitual, de maneira que podemos reconhecer não apenas modificações das condições locais, mas quando fica aparente uma modificação geral inerente ao organismo como um todo”. Atualmente não podemos mais nos contentar com essa definição, como demonstra Rößle,^6^ que espera que a “sífilis constitucional” e coisas semelhantes sejam definitivamente eliminadas.

Uma terceira concepção entende que se pode falar de características constitucionais *quando* essas características permitem excluir completamente uma determinada direção, e se apresentam sub- ou sobrevalorizadas em relação à média, para que o indivíduo em questão, de maneira geral, seja visto como tendo uma constituição “forte” ou “fraca” em cada aspecto. Tal classificação geral de unidades orgânicas, no entanto, como Toenniessen^7^ destacou, perde seu fundamento com os estudos sobre hereditariedade, pois reconhece apenas modificações de fatores hereditários pontuais, circunscritos, cujas variações correspondem a funções parciais bem definidas.

Essas três concepções do caráter da constituição ainda estão em muitas mentes e reaparecem aqui e ali nos escritos; de maneira geral, no entanto, podem ser consideradas superadas. O que resta é nada menos que um todo coerente. As definições variam uma com relação às outras, pois, para um, a constituição é um *fluxo* [*Ablauf*],^8^ ma *forma de reação*, ou seja, algo dinâmico; já outro a vê como uma *capacidade de reação* ou uma *estrutura* [*Anlage*], ou seja, algo potencial; já a um terceiro pode parecer uma *soma de características* ou mesmo um *complexo de sintomas*, algo totalmente estático. Se olharmos mais de perto, sem ficar presos às formulações verbais dessas definições, mas tentando compreender seu sentido, muitas das contradições se dissolvem; é possível reconhecer ao menos o contorno de um núcleo comum e achar que os avaliadores individuais se diferenciam mais na maneira de se expressar do que em suas concepções, que as diferenças são mais de natureza formal do que essenciais. *De forma relativamente consistente, o caráter da constituição é visto atualmente como uma forma específica de resposta ao estímulo*; essa concepção surge com Hüeppe^9^ e, em especial, com Martius; no entanto, alguns ignoram que a constituição *não pode ser definida propriamente como uma resposta a estímulos*, mas apenas como um estado, ou uma forma de configuração do corpo ou de suas partes, da qual decorre, resulta ou tem algum tipo de correlação a suposta resposta aos estímulos. Apenas isso corresponde inteiramente à etimologia da palavra constituição = compilação [*Zusammenstellung*], composição [*Zusammensetzung*], constituição [*Verfassung*], condição [*Beschaffenheit*], ordenamento [*Anordnung*] ou organização [*Einrichtung*] – e é o exigido pelo uso da linguagem.

O fato de que a concepção da matéria viva não leva em conta seu estado não altera o meu ponto de vista. Nela não se encontra nada estável, mas uma interminável sequência de operações em andamento, como ressaltou Grote.^10^ É certo que cada entidade viva apenas um estado de equilíbrio dinâmico, como, por exemplo, uma cachoeira ou uma chama, mas as condições para a configuração temporária desse equilíbrio são – graças a diversas circunstâncias (princípio de integração, “modificações permanentes” etc.) – suficientemente estáveis a ponto de permitir ao médico [o uso de] expressões como “estado corporal” [*Körperzustand*] ou “constituição corporal” [*Körperverfassung*].

O que mais atrapalha o entendimento é outra coisa: a diferença de opiniões *sobre as fontes* do que se denomina constituição. Em outras palavras, *um grupo de pesquisadores, em oposição a outro, acredita ter reservado para si a posse [exclusiva] da denominação da descarga da herança genética, no sentido estrito da palavra*. Seria possível dizer que se trata, nesse caso, apenas de uma questão secundária, de nomenclatura; basicamente seria indiferente se dividimos a constituição em decorrente da hereditariedade, ou seja, genotípica ou idiotípica,^11^ e em adquirida ou paratípica [fim da página 817] ou se denominamos a primeira constituição e a última com outro nome.^12^ *Porém não é assim*; essa divergência que é, antes de tudo, puramente classificatória, está associada ao confronto de duas concepções estreitamente ligadas à natureza das coisas, e das quais uma deve ser descartada. É o que se tentará fazer a seguir.

Em palestras proferidas na Sociedade Alemã para Higiene Racial, em 1913, o *anatomista* vienense Tandler^13^ fez a diferenciação entre constituição de causa hereditária e uma “condição” causada pelo ambiente, ou paratípica. Sobre isso, a seguinte citação é especialmente pertinente: “*As características individuais do soma* [corpo celular] *determinadas no momento da fecundação representam sua constituição. Com isso está implícito que eu entendo como constituição as variações individuais; depois de deduzidas as qualidades da espécie e da raça, o que resta são as características morfológicas e funcionais do novo indivíduo. A constituição*, entendida nesse sentido, *não é mais alterada por estímulos*, mesmo que esses atuem diretamente no soma do indivíduo, *ela é o destino* [*fatum*] *somático do indivíduo*. Se examinamos a composição corporal de uma pessoa a partir dessas premissas, somos obrigados a considerar a constituição algo dado, imutável. E tudo aquilo que frequentemente é designado como constituição herdada não pode ser subsumido ao conceito de constituição. *Em um indivíduo, o que pode ser alterado por influência do meio não é nunca sua constituição, e sim sua condição*”.

O importante aqui seguramente já havia sido defendido ocasionalmente *antes* de Tandler – ao menos por Mathes;^14^ ao que recentemente ele mesmo e Toennissen atribuíram grande importância. Em sua obra, que se tornou um clássico das concepções sobre a constituição no campo da ginecologia e que provavelmente foi o primeiro trabalho feito nesta acepção, *O infantilismo, a astenia e suas relações com o sistema nervoso* (Berlin, 1912), Mathes caracteriza a constituição ou compleição como sendo determinada primariamente pela constituição das células germinativas dos pais; na vida individual ela não sofreria modificações, não seria influenciável por efeitos externos, no sentido da doutrina de Weismann,^15^ da continuidade do germoplasma do patrimônio familiar.

Essa concepção de constituição e sua diferenciação da concepção de condição sem dúvida tem, em primeiro lugar, muitas coisas marcantes, o que fez com que muitos pesquisadores experientes a adotassem – alguns “com entusiasmo” e sem reservas, e outros com determinadas alterações recalcitrantes ou até mesmo, no fundo, apenas porque já se havia radicado na literatura, devendo por isso ser mencionada (Grote).

No que diz respeito às modificações, [esses autores] em parte, ampliaram a definição de Tandler. Löhlein,^16^ Toenniessen, Rößle, Siemens ressaltam – certamente com razão – que não existe motivo para suprimir as qualidades específicas e de raça do conceito (clínico) de constituição, uma vez que as disposições de espécie e raça formam partes essenciais do que deve interessar ao médico, com relação à constituição de seu paciente, uma vez que são determinantes para a origem e o decurso da doença. Além disso, é conveniente ressaltar, como fazem Lenz, Grote e outros, que à constituição pertencem não apenas características do soma, mas também da psique. Aliás, talvez Tandler pretendesse opor o “soma” apenas ao germoplasma, e não, como muitos parecem ter entendido, à psique.

Por outro lado, a definição de Tandler foi estruturada de forma mais restrita, aparentemente mais por equívoco do que de propósito. Negligenciamos facilmente o fato de que o *próprio Tandler nunca identificou a constituição com o conceito de genótipo*, ou seja, com sua composição hereditária, no sentido mais estrito do termo, como fizeram depois Löhlein e Hart.^17^ Sua formulação, como mencionada, permite ver que ele não aceita (explicitamente), por exemplo, a presença de configurações genéticas heterogaméticas recessivas e não descarta – ao menos não expressamente – a preconcepção de danos genéticos paracinéticos. J. Bauer, que considera efetivamente comprovada a ocorrência de tais danos genéticos (por doenças, venenos etc.), vê aí um momento em que a constituição se apresenta isenta das influências dos processos hereditários. Essa independência é ressaltada também por Toenniessen, que entende ser apenas o citoplasma das células germinativas atingido pela blastoforia paracinética, à qual as qualidades hereditárias da massa germinativa^18^ aderem. No entanto, em oposição a Bauer e diferindo da formulação de Tandler, [Toenniessen] entende tais momentos, decorrentes (ou seja, parafóricos – o Autor)^19^ do dano genético, como *não* constitucionais.

Isso serve para ilustrar que a delimitação feita por Tandler, e que, ao menos conceitualmente, se impunha como sendo absolutamente nítida, pode dar lugar a alguma dúvida. Toenniessen a considera absolutamente nítida porque no ser humano não existe hereditariedade de caracteres adquiridos. É de se notar, em contrapartida, que Tandler é declaradamente lamarckista; ele considera possível, portanto, o que hoje é uma condição amanhã ser constituição,^20^ e, para seu precursor Mathes, essa modificação pode ocorrer também dentro de um mesmo indivíduo, ponto ao qual retornaremos adiante. Também J. Bauer salienta que a diferenciação entre caracteres herdados constitucionais e condicionais – mesmo em termos puramente teóricos – nem sempre pode ser feita com exatidão.

Gostaria de demonstrar isso com um exemplo específico que Hart empregou em uma relação semelhante. Entre os fatores mais importantes na determinação da constituição corporal estão as modificações associadas ao envelhecimento fisiológico. Serão essas constitucionais ou condicionais? Seguramente todo o processo de desenvolvimento individual, com seus trechos ascendentes e descendentes, está essencialmente predeterminado no plasma germinativo. “A curva de crescimento da vida inteira é hereditária” (Friedenthal).^21^ Lembremos, por exemplo, da longevidade familiar. As características pessoais da senescência (de acordo com o grau e o momento em que ocorrem) seriam, portanto, constitucionais. Por outro lado, conhecemos as relações das alterações devidas ao envelhecimento com a formação de paraplasma, com a diferenciação, com a carga funcional e com as condições ambientais. A divisão conceitual entre constituição e condição, no sentido que lhe dá Tandler, repousa assim sobre os problemas mais difíceis e ainda não resolvidos da mecânica do desenvolvimento,^22^ no que diz respeito à diferenciação funcional e autodiferenciação.

*As objeções que me parecem existir sobre a divisão proposta por Tandler*, no entanto, são de espécie completamente diferente. Temos que nos perguntar, sobretudo, quais “características individuais do soma” são realmente “determinadas no momento da fecundação”, conforme propõe Tandler para as características constitucionais. Em minha opinião: nenhuma! Um “soma”, no sentido que lhe é atribuído habitualmente, não existe de modo algum nesse momento, e, assim que começa a existir, em seu vir a ser já começam a tomar parte momentos peristásicos (ambientais). *O genótipo em si não carrega qualidades somáticas* ou *“o gene em si não é causa de nada”* – como menciona Toennissen citando Kraus,^23^ com o adendo de que os fatores hereditários são apenas as fontes dos estímulos (talvez fosse melhor dizer os conversores dos estímulos – o Autor), que o citoplasma, *como o órgão executor*, incita seu desempenho específico. O genótipo ou o idiótipo é mera “moldura” constitucional, ele apenas define uma “soma de possibilidades de reação” (Lenz, 1912); a *verdadeira configuração* do soma dentro desse enquadramento, a determinação de todas as dimensões morfológicas e funcionais na zona de variação hereditária, é decorrente de influências extra-hereditárias, da paracinese. O parátipo resulta de variações não hereditárias ou paravariações, e da interação dessas com o idiótipo resulta o fenótipo. Apenas este último é objeto da avaliação médica, do exame funcional. *Se a constituição deve permanecer ou mesmo tornar-se um conceito clinicamente útil, então ela deverá em primeiro lugar, de acordo com o anseio geral, ser diretamente comprovável no objeto,* [fim da página 818] *ser numericamente apreensível e mensurável em suas inúmeras manifestações; e isso ela poderá ser apenas se for reconhecida como associada ao fenótipo e como específica dele*. Uma constituição puramente genotípica seria sempre uma miragem, um conceito com o qual o médico, em nove entre dez casos, não saberia por onde começar. Se, como já foi dito, a constituição for considerada um estado caracterizado, de maneira geral, por determinada forma de reação, então ela tem que estar associada inevitavelmente a uma capacidade direta de reação a uma existência real, ou seja, estar ligada ao fenótipo, e não ao genótipo.

Imaginemos dois gêmeos univitelinos, isto é, dois indivíduos genotipicamente idênticos; um deles, em razão de um estilo de vida e treinamento corporal apropriados, torna-se um homem musculoso, enquanto o outro, sob influências menos favoráveis do ambiente, tende para o extremo oposto da amplitude da faixa de desenvolvimento que lhe é determinada pela hereditariedade. Em caso de uma decaída, por exemplo, quando houver a possibilidade de um esgotamento, deverá o médico considerar a determinante prognóstica “constituição” de ambos indivíduos equivalente – como preconiza Tandler – ou não?!

Minha opinião é, portanto: se quisermos evitar que a constituição desapareça do universo dos conceitos médicos, então temos que *defini-la como algo que é assim ou assado, e não como algo que, dependendo das condições, pode tomar uma ou outra forma*. Para a clínica são significativas não tanto *as possibilidades anteriores de reação,* mas muito mais *determinadas capacidades e formas de reação*. Do meu ponto de vista – contrário ao de Tandler –, as características individuais definidas no momento da concepção, de maneira geral, *não* são o destino somático do indivíduo. Podemos atribuir a esse, provavelmente, certos casos extremos, relativamente raros, nomeadamente as doenças hereditárias graves, no sentido mais estrito das palavras. No que diz respeito à constituição, porém, ao médico interessam também outros casos que não se desviam, ou se desviam muito pouco, da normalidade e que ocorrem em maior número. Quem quiser reconhecer a constituição no genótipo, a mim parece alguém que se esforça por ler um “relógio” do qual pode ver apenas a mola tensionada, mas não o sistema de ancoragem do pêndulo que regula a velocidade da liberação da tensão e nem os ponteiros ou o mostrador.^24^

*É quase desnecessário enfatizar o quanto seria desejável e significativa, do ponto de vista aqui defendido, a pesquisa genealógica do genótipo, e saber que isso já pode ser feito em seres humanos, com satisfatório sucesso, em casos com localização propícia.* ^25^

Conforme destacado, é em especial a clínica que, hoje mais do que nunca, não pode dispensar o conceito de constituição e tem que aprender a trabalhar com ele de forma sistemática e exata, para poder descrever o indivíduo. É melhor avaliar o que a teoria de Tandler tem a dizer sobre isso a partir de suas próprias palavras: “A verificação de uma constituição pode ocorrer durante um exame morfológico e funcional”. O autor mencionado utiliza há anos o tônus muscular como meio auxiliar de diagnóstico para determinar o pertencimento constitucional de um indivíduo; ele divide os seres humanos em hipertônicos, normotônicos e hipotônicos. Obviamente Tandler é obrigado a aceitar que existe um tônus condicional além do tônus constitucional, sendo que o primeiro depende de uma série de fatores externos. Aguardamos com ansiedade para saber quais métodos permitem ao autor ignorar esses componentes condicionais na alegada “comprovação constitucional”. Pois sobre isso ele continua: “Se abstrairmos essas variações de natureza condicional, cada indivíduo particular possui um tônus característico, que se manifesta das mais diferentes formas, mas sempre de maneira típica”. A coisa parece, portanto, muito simples: basta “abstrair” a componente condicional para reconhecer a constituição *sensu strictiori* [*sic*]. Naturalmente não se diz uma palavra sobre como conseguir operacionalizar essa abstração, embora esse seja o cerne da questão. Será possível mesmo denominar isso um procedimento para a avaliação do pertencimento constitucional, e, em caso afirmativo, será que esse procedimento preenche os requisitos mínimos de objetividade? O meu ponto de vista não pode ser mais bem ilustrado do que uma constituição puramente genotípica; apesar de ser um conceito claramente definido, não é objeto de estudos clínicos, não é mensurável nem pode ser determinado por meio de uma avaliação médica objetiva. Na tentativa de conseguir fazer essa determinação em um voluntário, o médico se depara com uma equação com duas incógnitas, ou seja, um problema insolúvel.

É interessante perceber que, gradualmente, também certo número de antigos partidários da teoria da constituição genotípica chegou a esse entendimento. Mathes fez recentemente um prognóstico ruim para a pesquisa sobre a constituição (no sentido que *ele* dá aos termos). Jamais será possível determinar a parte constitucional de um evento corporal da mesma forma como é determinado o teor ácido de uma solução por meio de titulação [análise volumétrica]. A constituição é “incorpórea”, e *uma patologia constitucional* (ao que me parece, é a isso que se dedica a excelente obra do autor citado) *é algo que nunca existirá, pois constituição é apenas um conceito*. Ela é, na verdade – e é assim que Mathes gostaria que fosse entendida –, se examinada em profundidade, um conceito estéril para o médico. Finalmente, Mathes chega à surpreendente sugestão: “Deveríamos levar em consideração a possibilidade de abandonar [o uso da] palavra constituição, tão discutida e sujeita a tantas interpretações, e colocar outra em seu lugar”, nomeadamente “constituição corporal” e “estado corporal”, de acordo com J. Bauer e com Toenniessen. Penso que não é necessário ser tão radical, basta substituir a definição genotípica, parcial e inadequada, pela mais antiga, a fenotípica.

*Uma importante nota de pé de página a sua definição de 1912 deixa perceber que já então Mathes achava o conceito genotípico por demais restrito. Ali se pode ler que Martius também se refere a doenças constitucionais adquiridas e elege, como paradigma desses casos, a sífilis. Martius tem razão, pois a sífilis acomete “uma parte integrante da constituição, o sistema generativo”. “O portador da sífilis está constitucionalmente doente porque ele, como portador individual do plasma germinativo permanente, o lesionou com seu adoecimento. Não levando em conta que a presumível lesão do plasma germinativo provavelmente não ocorre regularmente, as divergências com as definições do conceito, como compreendidas pelo autor, são inevitáveis. Para Mathes, a constituição é determinada pela natureza das células germinativas dos pais. Que uma idiovariação do plasma germinativo possa influenciar retroativamente a constituição corporal do indivíduo da qual se origina, para mim é uma coisa inédita. O próprio Mathes parece ter alterado posteriormente sua concepção sobre isso; ele afirma (1920) que o plasma germinativo não poderia ser modificado pelas forças que afluiriam de seu próprio corpo durante sua conformação nem de forma substancial nem permanentemente.*

As dificuldades criadas por dois outros seguidores de Tandler, J. Bauer e Hart, a partir de sua definição, são demonstradas por Siemens, cujas considerações mencionamos a seguir.

Também para Toenniessen a teoria, da qual em determinado momento se declarou partidário, parece ter necessitado alterações. Em seu artigo mais recente que, de maneira geral, dá sequência à linha de pensamento de uma coletânea muito informativa, publicada três anos antes, ocorre ao especialista, na justificação da definição genotípica, uma nova intervenção: é preciso admitir que uma série de características fenotípicas possui, ao mesmo tempo, componentes hereditários e adquiridos. Sobre isso nos informa exatamente o seguinte texto: os fatores hereditários concretizados não devem ser vistos como algo absoluto; apenas nos casos mais raros uma característica é determinada apenas hereditariamente, em geral, é ao mesmo tempo influenciada pelo estímulo do meio ambiente; ela flutuaria dentro dos limites hereditários em um determinado valor médio. Se dermos razão a Tandler e Bauer e considerarmos apenas as características puramente hereditárias constitucionais, e tudo o que se afasta do tipo hereditário ideal condicional, então “*o conceito de constituição se reduziria a um âmbito tão pequeno que se tornaria irrelevante e supérfluo*”. (Compare o mencionado acima com a declaração, com o mesmo sentido, [fim da página 819] de Mathes.) Para ir ao encontro disso, a fim de, por assim dizer, preencher novamente o conceito esvaziado de constituição, o autor sugere *agora* denominar as variações (fisiológicas) do soma, resultantes de influências ambientais, “características somático-constitucionais” e as incluir no campo das reações hereditárias, ou seja [falha no texto] da constituição.^26^ Isso obviamente contradiz completamente a proposta de Tandler e vai mais ou menos na direção de formular o conceito de constituição de forma fenotípica. Isso, no entanto, Toenniessen não quer reconhecer; pois ele é de opinião que ao fenótipo também pertencem variações somáticas *patológicas* de origem hereditária (resultantes de influências ambientais *patogênicas*), e essas ele deseja excluir do campo constitucional – em contraposição às variações somáticas fisiológicas. O que complica o agrupamento feito de acordo com Toenniessen, e o diferencia de todos os outros, é, portanto, o fato de que ele traça uma linha reforçada entre a ação de influências ambientais causadoras de variações, porém não patogênicas, e as variações ambientais patogênicas (embora, provavelmente, umas se confundam com as outras – o Autor), considerando aquela ainda constitucional, mas esta última não mais; e sim pertencente à constituição corporal ou ao estado corporal (fenótipo). A constituição não seria alterada por esta última, apenas encoberta, tendo sua atuação dificultada.

Não sei se a perspectiva de Toenniessen vai encontrar seguidores, mas quis apresentá-la como comprovação de que também a sugestão de Tandler não irá contentar seus partidários no longo prazo.

Semelhante à nova definição de Toenniessen me parece ser a de Grote, que entende por constituição o somatório de todas as características morfológicas, funcionais e regulatórias do soma e da psique que são determinadas detalhadamente no momento da fecundação *e que se desenvolvem durante a formação do indivíduo*. O último adendo parece indicar a influência peristática e *também* levar em conta as coisas descritas por Toenniessen como características somático-constitucionais.

Em contraposição a essas duas definições, mais ou menos intermediárias, os outros autores reconhecem, como antes, de maneira aberta e sem reservas, o caráter fenotípico da constituição; pois entendem que esta se constitui por meio da atuação integrada de influências genotípicas e paratípicas, reconhecendo, ao lado de uma componente hereditária, também uma adquirida. Muitas vezes tal separação genética não é nem mesmo particularmente destacada, como, por exemplo, nas seguintes definições: “Capacidade de reação a influências externas” (Martius); “O tipo de reação do organismo depende de sua constituição [*Beschaffenheit*]” (Lubarsch);^27^ “Soma dos fatores endógenos^28^ de uma doença” (A. Schmidt);^29^ “Totalidade das circunstâncias organizacionais” (F. Kraus); “Totalidade do que oferece novas condições ao corpo” (Krehl);^30^ “Condições internas do sistema vital” (Brugsch).^31^ Outras vezes isso ocorre *expressis verbis*, como, por exemplo, na expressiva definição de Rößle: “Entendemos por constituição a composição [*Verfassung*] do corpo e de suas partes, formada por elementos inatos (melhor seria dizer hereditários – o Autor) e adquiridos, que podem ser reconhecidos pela maneira como eles ou elas respondem a estímulos ambientais”. Da mesma maneira, aproximadamente, de acordo com K.H. Bauer,^32^ ou de forma ainda mais abrangente, de acordo com F. Kraus: “Uma condição que é herdada ou adquirida pelo indivíduo, que pode ser analisada morfológica e funcionalmente e que também pode ser derivada do comportamento de certas funções individuais como do somatório do estado físico e mental e das características de desempenho, especialmente no que diz respeito à resiliência, resistência (susceptibilidade à doença), capacidade de rejuvenescimento e vitalidade do organismo”.

Tal como os [cientistas] mencionados acima, o Autor sempre defendeu a opinião de que a constituição e as anomalias constitucionais também podem ter componentes paratípicos; tal como os pesquisadores de hereditariedade mais atualizados, como Lenz e Siemens, ele gostaria de ver a constituição como um conceito dividido em condicionantes idiotípicas e paratípicas, que estariam presentes na constituição como o conteúdo resultante de experiências mistas. De um ponto de vista semelhante, Rößle, em especial, opôs-se resolutamente à proposta de Tandler e Friedrich Kraus, que no último quarto de século [passado] transitou da “elevada” concepção do “cansaço como medida da constituição” para a “patologia da pessoa”, e investigou incessantemente o problema da constituição – não só com todos os instrumentos das ciências naturais, mas também com os da filosofia –, chegando à conclusão de que o melhor e mais consistente nas declarações de Martius é a referência à irritabilidade modificada como sendo comum aos estados constitucionais, afirmando que as definições do que seria constitucional, entendido como um modo especial de reação anormal, *devem* incluir o genótipo *e* as modificações adquiridas. A percepção de Tandler (que Kraus conecta com sua teoria do “todo original”) compreende apenas uma parte do que foi e é atualmente denominado constitucional.

*Nem Tandler – ao contrário da opinião de alguns – identificou a constituição com o idiótipo (ver acima, p.818), tampouco ela deve ser totalmente equiparada ao fenótipo. Siemens explica que este último é um conceito teórico-biológico, e o primeiro, clínico-prático; a constituição só diz respeito ao fenótipo quando tem, para o médico, significado clínico, ou seja, quando fornece ao médico informações sobre a disposição de seu paciente. Essa mudança é incorporada por esse autor a sua definição de constituição, o que me parece ir um pouco longe demais – porque essas conclusões são, de início, ainda fortemente subjetivas, instáveis e dificilmente confiáveis. A mesma ideia, talvez um pouco mais moderada, pode ser encontrada em Lenz, que considera constituição, de maneira geral, a condição corporal em relação a sua “probabilidade de preservação” [Erhaltungswahrscheinlichkeit] ou, o que dá no mesmo, a sua resistência. A partir dessas premissas, coloca muitas questões gerais e específicas de grande relevância, com lucidez e clareza exemplares.*

A principal objeção contra a definição fenotípica é o caráter instável associado à constituição. Este é entendido, de maneira geral – ou, ao menos, assim parece –, como algo mais estável. Não me parece que isso esteja resolvido. Quer me parecer que, em lugar de pressupor flutuações espontâneas periódicas da constituição e da disposição, causadas por influências geofísicas, que surgiriam como consequência de doenças ou seriam provocadas aleatoriamente por tratamentos diversos, seria mais recomendável seguir as numerosas medidas e recomendações da medicina, de todos os tempos e lugares.

A esta exposição sobre a constituição pode ser acrescentada alguma coisa sobre anomalias e doenças constitucionais.^33^ Em minha opinião, aqui é melhor partir do conceito de doença e desvio patológico de Lenz. Esse autor se guia pela “probabilidade de preservação”. Desvios que causam diminuição da “probabilidade de preservação” de seus portadores são patológicos, e o organismo está em estado patológico quando alcança os limites de sua capacidade de adaptação. Se tais circunstâncias forem decorrentes de especificidades constitucionais do corpo, temos uma anomalia ou doença constitucional. O mesmo ocorre quando empregamos a noção de normalidade de Grote. Ao contrário da “norma ideal”, sempre puramente subjetiva, ao contrário da fictícia “norma estatística” (que não existe, em realidade, em nenhum espécime), aparece um tipo diferente de norma real e racional, no sentido biológico e clínico. Essa norma surge quando as expressões de vida de um indivíduo correspondem plenamente a suas necessidades biológicas, que resultam [fim da página 820] da coincidência de sua situação de vida externa^34^ com suas possibilidades de desempenho fisiológico. Para tal “autoconformidade” [*Sichselbstentsprechen*], “autocongruência” [*Insichkongruentsein*], Grote utiliza a expressão *responsividade* [*Responsivität*]. Se o desempenho biológico do indivíduo é suficiente para sua própria preservação, se – e por quanto tempo – permanece *completamente* responsivo, pode ser considerado normal. De acordo com isso, um indivíduo constitucionalmente *anormal* é aquele cuja condição física o torna vulnerável a um comportamento irresponsável, constitucionalmente *patológico*, quando esse indivíduo, temporária ou permanentemente, não consegue suficientemente recuperar sua “responsividade” por meio de adequação morfológica ou funcional.

As anomalias constitucionais podem provir diretamente das definições fenotípicas de constituição acima apresentadas. As patologias constitucionais surgem efetivamente no solo das anomalias constitucionais, pois as influências ambientais agem continuamente sobre a resposta anormal ao estímulo, ameaçando temporária ou permanentemente a adaptação do indivíduo. *Um* caso-limite é quando essas influências ambientais permanecem invisíveis, quando não vão além do âmbito dos estímulos fisiológicos; nessas circunstâncias, são consideradas doenças hereditárias (no sentido mais estrito da palavra).

**Figure:** Legenda para o dispositivo sobre as relações mútuas de constituições normais, anomalias constitucionais e patologias constitucionais de origem idiotípica e paratípica. Em 1 – Área das doenças hereditárias stricto sensu (p.ex., distrofia muscular progressiva). Em 2 – Área dos danos “idiodispositivos” de origem combinada (p.ex., escrófula, raquitismo). Em 3 – Área das doenças constitucionais puramente ectógenas (p.ex., “doença do trigo sarraceno”, caquexia por chumbo). Em 4 – Área das anomalias constitucionais puramente hereditárias (p.ex., hemofilia). Em 5 – Área das anomalias constitucionais de origem mista (p.ex., astenia). Em 6 – Área das anomalias constitucionais puramente ectogénicas (p.ex., estado de eunuco após a castração, certas anafilaxias e idiosincrasias adquiridas).

[Fonte: Pfaundler, 22 abr. 1922, p.821]

*Naturalmente, os limites indicados não são considerados muito rígidos e nem absolutos. Norma e anomalias, assim como anomalias e patologias, fluem de uma para outra, o que explica desvios subjetivos nas alocações.*

As componentes idiotípica e paratípica da patogenia comportam-se geralmente de forma recíproca de acordo com suas dimensões. Outro caso-limite é que tanto a anomalia constitucional quanto os danos manifestos são de origem ectogênica.

Para fins didáticos, vamos tentar representar graficamente as relações mútuas mencionadas no título do presente trabalho. Uma representação gráfica razoavelmente satisfatória se encontra no diagrama adjacente. Em um disco, cuja circunferência define o limite de conservação e que inclui, portanto, tudo o que é viável, aparecem dispostos de forma concêntrica: um núcleo formado pelas *constituições normais*, mais distantes do limite da “probabilidade de preservação”; depois, aproximando-se do limite da capacidade de resposta, a área das *anomalias constitucionais* ^35^ e, finalmente, mais além, no limite da preservação, as *doenças constitucionais*. *Um* dos polos do disco corresponde ao momento inicial idiotípico, o polo oposto ao paratípico; os arcos entre eles abrangem as origens, que interagem em uma relação variável. Logo em seguida aparece a posição de diferentes objetos nesse *Orbis morborum*, mais ou menos como a legenda do diagrama apresenta, com alguns exemplos. Nas anomalias constitucionais, é conveniente distinguir certos grupos de acordo com o seguinte esquema:

**Tabela:** 

Anomalias constitucionais	Variante individual preponderantemente morfológica	Variante individual preponderantemente funcional
Bastante espalhada no organismo	Anomalias no *habitus* ou variações múltiplas	Diátese ou disposição para doenças em geral
Mais localizada	Malformações	Vulnerabilidade em órgãos e sistemas (suscetibilidade)

[Fonte: Pfaundler, 22 abr. 1922, p.821.]

Em todos esses grupos encontramos anomalias herdadas bem como adquiridas; os exemplos a seguir devem servir como evidência; naturalmente é preciso fazer a ressalva de que a teoria dominante na atualidade pode apresentar algumas modificações, em um ou outro ponto.

**Tabela:** 

Anomalias constitucionais	De origem preponderante ou puramente idiotípica	Origem combinada	De origem preponderante ou puramente paratípica
Anomalias no *habitus* ou variações múltiplas	Formações múltiplas de exostose cartilaginosa, condrodistrofia	Diversas formas de infantilismo, obesidade etc.	*Status strumiprivus*
Malformações	Sindactilia	Varizes, vegetações adenoides	Estrangulamentos amnióticos
Diáteses	Diátese exsudativa	Espasmofilia, vagotonia, neuroartritismo	Suscetibilidade pós-morbosa a infecções (a tuberculose, difteria)
Vulnerabilidade em órgãos	Aquilia gástrica, miopia	Albuminúria lordótica, algumas constipações habituais, cardiopatia na adolescência	Alterações cardíacas devidas a doenças na tireoide ou traqueia [*Kropfherz*]

[Fonte: Pfaundler, 22 abr. 1922, p.821.]

Com bastante lógica, Hart, Siemens e outros pesquisaram recentemente as *relações entre constituição e disposição*. Existiria uma reciprocidade entre elas, ao contrário do preconizado por Martius – para quem os dois termos não estão de modo algum na mesma linha: a primeira seria autônoma, a segunda, relativa e, portanto, específica. Dever-se-ia tomar cuidado ao encetar discussões biológico-científicas com a utilização do conceito de constituição, empregado com diferentes significados e nuanças, ao contrário do termo disposição, que não abrange nenhum conceito teórico-científico, apenas expressa determinado empirismo clínico. A patologia constitucional se tornará cada vez mais uma patologia da disposição, uma teoria da sintomatologia e da terapia de suscetibilidades patológicas de natureza bastante específica. Esse entendimento está ligado à definição da constituição como um “complexo de sintomas”, definição que não me parece muito feliz, e sim um tanto estreita. *Temos apenas que recordar a definição mais ampla de F. Kraus* (ver p.820), *para perceber rapidamente as relações de interdependência do termo em questão*: constituição é o conceito abrangente. [fim da página 821] Se a “condição” individual específica, mencionada por Kraus, em uma análise funcional se revelar uma redução no desempenho físico, no sentido de uma resistência reduzida a certo dano exógeno, então temos um caso especial de constituição ou de anomalia constitucional, que pode ser considerada uma mais elevada disposição ou suscetibilidade às doenças. Em muitos outros casos, isto é, em todos aqueles em que não se faz inicialmente uma análise funcional, mas sim morfológica, tal disposição não ocorre, mas, na melhor das hipóteses, há um complexo de sintomas (por exemplo, um estado tímico), ou seja, o que Siemens leva em consideração na moldura estreita de *seu* conceito de constituição. Ao pesquisar esse complexo de sintomas não se pode considerar dada uma disposição patológica. Pois importantes relações com tal disposição certamente serão investigadas, mas – *nota bene* – sem compromisso, e de nenhuma maneira o estabelecimento de uma síndrome constitucional típica pode ser vinculado, obrigatória ou facultativamente, à suscetibilidade a alguma patologia. Ela é *em si* interessante para o médico, pois pode estar atrelada a um correlato de ação funcional, que vai em direção oposta, um aumento da resistência [a doenças]. Uma anomalia constitucional pode atuar em ambas as direções; também é possível, e efetivamente ocorre, que a mesma variante pessoal prejudique o indivíduo em *um* sentido e o favoreça em *outro*. Com designações que contenham julgamentos generalizantes, como anomalia, degeneração, será necessário ser cuidadoso (Grote). Não seria desejável que a patologia constitucional se transformasse em uma disposição patológica, em especial da perspectiva de outras disciplinas que dela se ocupam, como, por exemplo, a antropologia.

Com isso concorda a opinião de Siemens sobre a questão da diátese, em seu excelente “Patologia constitucional e hereditária” [Konstitutions- und Vererbungspathologie]. Ele diz ser conhecido o fato de que a expressão diátese exudativa se refere a um quadro sintomático, um *status exsudativus* ou *exsudans*, e não, de forma nenhuma, a uma disposição específica a uma determinada patologia.

É admissível que, na reconstrução da antiga diátese inflamatória por parte dos pediatras, uma condição de características predominantemente morfológicas tenha sido descrita inicialmente como um complexo de sintomas; ou seja, algo que, de acordo com Siemens, seria uma patologia constitucional. Ocorreu, entretanto, uma mudança gradual, no sentido de que atualmente já não se costuma dizer “sintomas” de diátese exsudativa (ou outra diátese), mas sim “manifestações”, e que se tem reconhecido cada vez mais a natureza desse mal como sendo uma predisposição para a doença, que pode, muitas vezes, permanecer completamente latente em outros casos e circunstâncias, mas que se expressa nos indivíduos afetados sob certas influências externas, em geral inofensivas, sob a forma de reações parietais, catarrais ou inflamatórias, ou seja, desencadeando as manifestações acima mencionadas (cf., por exemplo, a apresentação do assunto no manual de Feers e em outros lugares). Não precisamos mais “desejar”, portanto, que haja uma identidade entre diátese e disposição, podemos tranquilamente constatá-la, mesmo sem levar em conta a etimologia; anteriormente uma patologia constitucional, a diátese exsudativa, ressuscitada tornou-se, na verdade, uma disposição patológica e, *desde então, é denominada “diátese” de pleno direito*; essa denominação não me parece supérflua e nem confusa, e sim bastante acertada, e poucos pediatras vão querer prescindir dela ou achar o nome *status exudans* excessivo ou até mesmo solicitar que esse estado seja retirado da classificação de patologia constitucional, como foi sugerido recentemente, pois ele foi claramente reclassificado como desordem metabólica (!?). Também sua relação com o estado tímico linfático em nada altera o verdadeiro caráter de diátese do pretenso estado.

## NOTAS

1 [Nota de R. Beraldo] Título original e local da publicação: PFAUNDLER, Meinhard von. Was nennen wir Konstitution, Konstitutionanomalie und Konstitutionskrankheit? *Klinische Wochenschrift*, v.1, n.17, 22 abr. 1922, p.817-822. Posteriormente publicada como capítulo na obra: RUDDER, B. (ed.). *Biologische Allgemeinprobleme der Medizin*. Berlin, Heidelberg, Spinger-Verlag, 1947.

2 [Nota de M. Pfaundler] De forma muito impressionante, por exemplo, por H.W. Siemens.

3 [Nota de R. Beraldo] Os trechos em itálico são do documento original.

4 [Nota de R. Beraldo] No decorrer do artigo, procurarei destacar nascimento e morte dos atores, o local de atuação (quando possível) e seus campos de atuação/interesse: Friedrich Martius (1850-1923); Alemanha; clínica médica.

5 [Nota de R. Beraldo] Rudolf Virchow (1821-1902); Alemanha; patologia.

6 [Nota de R. Beraldo] Robert Rößle (1876-1956); Jena e Berlim; patologia.

7 [Nota de R. Beraldo] E. Toenniessen (?-?); Erlangen; campo de atuação não encontrado.

8 [Nota de R. Beraldo] Optei por manter determinados termos no original em alemão porque fazem parte de um conjunto terminológico frequentemente acionado pelos atores médicos que discorreram a respeito do problema das constituições.

9 [Nota de R. Beraldo] Ferdinand Hüeppe (1852-1938); Wiesbaden e Praga; higiene e bacteriologia. Sobre a influência dos trabalhos de Hüeppe em Martius, ver Timmermann (1996).

10 [Nota de R. Beraldo] Louis R. Grote (1886-1960); Alemanha; internista.

11 [Nota de M. Pfaundler] Utilizamos as seguintes expressões, tiradas de Johannsen, Lenz e Siemens: Genótipo = idiotipo para o estado da configuração genética, constituição hereditária. Gen = Jd para unidades hereditárias. Paratipo para a soma das aquisições não hereditárias de um ser vivo. Fenótipo para o quadro sintomático, a totalidade das características hereditárias e adquiridas expressas em um indivíduo. Idiovariações para variações causadas por uma modificação no idioplasma. “Paravariação” [*Paravariation*] para variações não hereditárias, originadas por condições ambientais. Perístase para ambiente, meio, situação de vida. Paraforia para a ação secundária de “paravariações” sobre gerações subsequentes. [Nota de R. Beraldo] Os autores mencionados são: Wilhelm Ludvig Johannsen (1857-1927), botânico e fisiologista; Hermann Werner Siemens (1891-1969), dermatologia em Leiden; Fritz Lenz (1887-1976), higiene racial em Berlim.

12 [Nota de R. Beraldo] A nota anterior exemplifica o tipo de discussão médico-teórica que permeava o tema da *Konstitutionsforschung* naquele contexto, ou seja, pensar o papel dos fatores ambientais e hereditários para a formulação de concepções de saúde e doença.

13 [Nota de R. Beraldo] Julius Tandler (1869-1936); Viena; anatomia.

14 [Nota de R. Beraldo] Paul Mathes (1871-1923); Innsbruck; anatomia e ginecologia.

15 [Nota de R. Beraldo] August Weismann (1839-1914); Alemanha; biologia. Seus interesses de pesquisa giraram em torno do campo da embriologia e hereditariedade. O trecho referido diz respeito à teoria de Weismann segundo a qual as células germinativas (células do ovário e dos testículos) tendem a passar incólumes pelas transformações ocorridas em outras partes do organismo. Os efeitos de tais mudanças não podem ser transmitidos de pais para filhos. Consultar Allen (2017, p.33-34).

16 [Nota de R. Beraldo] Max Löhlein (1877-1921); Alemanha; patologia. Seu trabalho sobre os conceitos de “condição” e “constituição” era frequentemente citado na bibliografia correlata ao tema.

17 [Nota de M. Pfaundler] Por exemplo, Hart: peculiaridades do organismo, que no momento da união de óvulo e espermatozoide são produzidas a partir do material genético dos ascendentes. [Nota de R. Beraldo] Carl Hart (1876-1922); Berlim; anatomia patológica. O nome de Hart é central para a discussão sobre até que ponto a constituição individual seria manifestação do genótipo ou do fenótipo. Carl Hart, Toenniessen e Julius Bauer (1887-1979, clínico e endocrinologista vienense), citados no mesmo parágrafo, estão envolvidos criticamente com a definição de constituição como “destino somático do indivíduo”. Tal definição remonta a Paul Mathes e Julius Tandler, que, desde o começo da segunda década do século XX, articularam essa forma de ver a constituição individual. Em Pfaundler, nota-se a crítica da falta de utilidade prática (na clínica) desse conceito genotípico da constituição. Ver Timmermann (1996, p.26-27).

18 [Nota de M. Pfaundler] O monopólio da hereditariedade do núcleo ainda é discutido por outros [autores].

19 [Nota de R. Beraldo] A ocorrência “o Autor” está presente no original e se refere a interpretações singulares feitas pelo próprio Pfaundler no diálogo com seus interlocutores.

20 [Nota de M. Pfaundler] Comparar com Lenz, em sua análise sobre as “Palestras” de Bauer. *Münchener medizinische Wochenschrift*, 1922, n.7.

21 [Nota de R. Beraldo] Refere-se a Hans Wilhelm Carl Friedenthal (1870-1942); Alemanha; antropologia e fisiologia.

22 [Nota de R. Beraldo] A tradição de pesquisa sobre a “mecânica do desenvolvimento” [*Entwicklungsmechanik*] remonta ao final do século XIX e início do XX, com destaque para Wilhelm Roux (1850-1924) e Jacques Loeb (1858-1957), nomes importantes da embriologia e fisiologia experimentais alemãs. Pautar os fenômenos vitais sob interpretações físico-químicas, moleculares e cromossômicas estava dentro do horizonte de atuação do experimentalismo alemão daquele contexto. Portanto, podemos ver que, internamente à própria discussão sobre os aspectos genotípicos, hereditários, fenotípicos, ambientais e condicionais do conceito de constituição, existe toda uma discussão a respeito de explicações mecanicistas e reducionistas dos fenômenos biológicos. A esse respeito, ver Beraldo (2021), Allen (2017), Canguilhem (2012).

23 [Nota de R. Beraldo] Friedrich Kraus (1858-1936); Berlim; clínico. Kraus dispensava a parcialidade da definição do conceito de constituição, ou seja, atribuído apenas ao genótipo.

24 [Nota de R. Beraldo] A metáfora das “partes” *versus* “todo” desempenha papel central nessa altura do artigo de Pfaundler, certamente influenciado por Kraus, a quem tece elogios.

25 [Nota de R. Beraldo] No documento original esse trecho, assim como outros, aparece com uma letra de tamanho menor do que a do restante do texto, significando uma espécie de destaque. Decidi colocar no formato itálico.

26 [Nota de M. Pfaundler] As variantes de senescência, acima mencionadas, cuja classificação/afiliação é questionável, devem pertencer a essa categoria.

27 [Nota de R. Beraldo] Otto Lubarsch (1860-1933); Alemanha; patologia. Lubarsch estava na “lista” dos clínicos que relativizavam a interpretação do conceito de constituição baseada somente no genótipo.

28 [Nota de M. Pfaundler] Os fatores endógenos são, evidentemente, de origem mista, não só hereditários, mas também adquiridos; basta pensar na caquexia da tireoide, no aumento pós-infecção da disposição em comparação com outros danos ectogênicos etc.

29 [Nota de R. Beraldo] Dados não encontrados.

30 [Nota de R. Beraldo] Ludolf von Krehl (1861-1937); Alemanha; clínica e fisiologia. O nome de Krehl está ligado à fisiologia patológica, antropologia médica e psicossomática. Entre seus assistentes de destaque em Heidelberg estava Viktor von Weizsäcker (1886-1957), um dos promotores da medicina psicossomática, campo ao qual vinculam-se debates sobre corpo-mente e suas relações com as patologias.

31 [Nota de R. Beraldo] Theodor Brugsch (1878-1963); Halle; medicina interna. Pertencia à linha segundo a qual o conceito de constituição não deveria ser reduzido ao genótipo. Brugsch e Gustav von Bergmann (1878-1955) conformaram a escola de R. Kraus de pesquisa constitucional na Charité de Berlim. Em suas pesquisas referiam-se à ideia de totalidade da pessoa, *Personalismus*, bem como “patologia funcional”. Foi nessa mesma clínica que o italiano Nicola Pende (1880-1970) fez seis meses de especialização em 1914. Para as apropriações da escola alemã e italiana na medicina brasileira, ver, respectivamente, Beraldo (2021) e Vimieiro-Gomes (2012, 2015, 2016). Especificamente a respeito da obra de Brugsch, Kraus e Bergmann, ver Beraldo (2021) e Timmermann (1996).

32 [Nota de R. Beraldo] Karl-Heinrich Bauer (1890-1978); Breslau; cirurgia.

33 [Nota de R. Beraldo] A partir daqui há uma mudança no conteúdo da discussão e Pfaundler passará a dissertar sobre anomalias e doenças constitucionais.

34 [Nota de M. Pfaundler] Essa situação de vida externa provavelmente não é considerada particularmente benevolente nem particularmente prejudicial. As necessidades e dificuldades que caracterizam o meio ambiente, ao qual o organismo deve permanecer responsivo e adaptável, parecem ser um ponto ainda fraco nessa definição. Lenz leva isso em conta, referindo-se sempre explicitamente ao conceito de doença como relativo.

35 [Nota de M. Pfaundler] O que se entende aqui são apenas as anomalias constitucionais com uma probabilidade de preservação abaixo do máximo.

## References

[B1_pt_add] SIEMENS (1921). Einführung in die allgemeine Konstitutions- und Vererbungspathologie.

[B2_pt_add] MARTIUS (1914). Konstitution und Vererbung.

[B3_pt_add] VIRCHOW citado em RÖßLE.

[B4_pt_add] RÖßLE (1921). RUDOLF VIRCHOW und die Konstitutionspathologie. Münchener medizinische Wochenschrift.

[B5_pt_add] TOENNIESSEN (1921). Vererbungsforschung und innere Medizin. Ergebnisse der innere Medizin und Kinderheilkunde. 17. 1919.

[B6_pt_add] TANDLER (1913). Konstitution und Rassenhygiene. Zeitschrift für angewandte Anatomie und Konstitutionslehre I.

[B7_pt_add] GROTE (1921). Grundlagen ärztlicher Betrachtung.

[B8_pt_add] LENZ (1921). Grundriss der menschlichen Erblichkeitslehre und Rassenhygiene.

[B9_pt_add] MATHES (1912). Der infantilismus, die Asthenie usw.

[B10_pt_add] LÖHLEIN (1918). Die Begriffe, Konstitution und Disposition. Medizinische Klinik.

[B11_pt_add] BAUER (1917). Konstitutionelle Disposition zu inneren Krankheiten.

[B12_pt_add] HART (1918). Konstitution und Disposition. Berliner medizinische Wochenschrift.

[B13_pt_add] KRAUS (1919). Die allgemeine und spezielle Pathologie der Person.

[B14_pt_add] Bauer K.H. Über den Konstitutionsbegriff. Zeitschrift für Konstitutionslehre.
